# FWNet: Semantic Segmentation for Full-Waveform LiDAR Data Using Deep Learning

**DOI:** 10.3390/s20123568

**Published:** 2020-06-24

**Authors:** Takayuki Shinohara, Haoyi Xiu, Masashi Matsuoka

**Affiliations:** Department of Architecture and Building Engineering, Tokyo Institute of Technology, Yokohama 226-8502, Japan; shinohara.t.af@m.titech.ac.jp (T.S.); xiu.h.aa@m.titech.ac.jp (H.X.)

**Keywords:** full-waveform lidar data, semantic segmentation, deep learning, supervised learning

## Abstract

In the computer vision field, many 3D deep learning models that directly manage 3D point clouds (proposed after PointNet) have been published. Moreover, deep learning-based-techniques have demonstrated state-of-the-art performance for supervised learning tasks on 3D point cloud data, such as classification and segmentation tasks for open datasets in competitions. Furthermore, many researchers have attempted to apply these deep learning-based techniques to 3D point clouds observed by aerial laser scanners (ALSs). However, most of these studies were developed for 3D point clouds without radiometric information. In this paper, we investigate the possibility of using a deep learning method to solve the semantic segmentation task of airborne full-waveform light detection and ranging (lidar) data that consists of geometric information and radiometric waveform data. Thus, we propose a data-driven semantic segmentation model called the full-waveform network (FWNet), which handles the waveform of full-waveform lidar data without any conversion process, such as projection onto a 2D grid or calculating handcrafted features. Our FWNet is based on a PointNet-based architecture, which can extract the local and global features of each input waveform data, along with its corresponding geographical coordinates. Subsequently, the classifier consists of 1D convolutional operational layers, which predict the class vector corresponding to the input waveform from the extracted local and global features. Our trained FWNet achieved higher scores in its recall, precision, and F1 score for unseen test data—higher scores than those of previously proposed methods in full-waveform lidar data analysis domain. Specifically, our FWNet achieved a mean recall of 0.73, a mean precision of 0.81, and a mean F1 score of 0.76. We further performed an ablation study, that is assessing the effectiveness of our proposed method, of the above-mentioned metric. Moreover, we investigated the effectiveness of our PointNet based local and global feature extraction method using the visualization of the feature vector. In this way, we have shown that our network for local and global feature extraction allows training with semantic segmentation without requiring expert knowledge on full-waveform lidar data or translation into 2D images or voxels.

## 1. Introduction

The airborne laser scanner (ALS) offers significant advantages for large-area observations in terms of speed and time-efficiency, compared to field surveying using a terrestrial laser scanner. However, manual operations to extract the spatial information from the data observed by ALS (ALS data) are costly and time-consuming. Automatic data processing methods for ALS data are necessary for practical applications. As in this review paper [1], most of the automatic processing for ALS data depends on 3D point-cloud-based methods. A typical method is a rule-based approach, such as classifying land cover using different thresholds for elevation, alongside statically calculated values [2]. A supervised machine learning approach is also used for point cloud classification [3]. At present, deep learning-based methods are widely used for 3D point cloud analysis. These 3D point clouds are defined as non-Euclidian data or spatially irregular data [4]. Therefore, most deep learning methods require conversion into Euclidian images or voxels [5,6]. To address this issue, PointNet [7] was developed to deal with the point cloud without a conversion process. PointNet can solve several tasks, including classification, semantic segmentation, and part segmentation. After the publication of PointNet, many studies in the computer vision field [5] have attempted to apply deep learning methods to ALS data [6]. For applications featuring deep learning-based 3D point cloud analysis, not only in topographic mapping, but also in many other fields, such as forest monitoring [8,9], power-line detection [10,11,12,13], and building detection [14,15]. However, most previous studies use limited geometric 3D point clouds without radiometric information, because such studies are highly dependent on open datasets without rich radiometric information [16,17].

As a radiometric observation instruments, full-waveform light detection and ranging (lidar)are widely used in ALS observations [18]. Full-waveform lidar record the entire reflected signal discretely. Some studies have shown that a full-waveform lidar data provides not only 3D point clouds but also additional information on the target properties such as the pulse width and the backscatter cross-section [19,20,21]. The shape and power of the backscatter of the waveform samples are related to the geometric and reflection characteristics of the reflective surface. Most full-waveform-based algorithms use this characteristic as a return signal [22,23]. For example, Mallet et al. (2011) [22] investigated the possibility of automatically discriminating three classes (buildings, ground, and vegetation) with an support vector machine (SVM) classifier using full-waveform lidar data. Digital Terrain Model (DTM) generation is performed by Gaussian decomposition on full-waveform signals, which increased the number of actual terrain points [23]. Full-waveform lidar data are beneficial for classification, because they provide valuable insights into the local organization of land use or land cover, which is detailed structural features about targets located along the transmission path of the laser signal. These algorithms described above for full-waveform lidar data analysis rely on handcrafted features [24] that are sent to rule-based algorithms or machine-learning algorithms. Maset et al. (2015) offered a data-driven alternative method, to solve the unsupervised classification task of waveform without calculating some handcrafted features, or converting into other data structures like image using self-organizing maps (SOMs) [25]. Additionally, Zorzi et al. (2019) [26] proposed a deep-learning based data-driven classification approach for full-waveform lidar data, which consist of point clouds with associated entire waveforms. In this method, classification tasks are divided into two steps. First, waveforms are classified using a data-driven feature (we simply refer to data-driven feature from deep learning model as a feature) extraction by the one-dimensional convolutional neural network (1D CNN). Next, the semantic segmentation task is solved using a fully convolution network (FCN) for 2D grid data, which includes height information from the point clouds and the class vector predicted by the trained 1D CNN in the first step. The main limitation of this method is that each waveform is learned individually in the first step. Furthermore, this method requires a large assumption that there is no occlusion, because it is projected onto a two-dimensional image. However, Zorzi et al. [26] demonstrated the necessity of using the spatial learning method for full-waveform lidar data via 2D FCN. Moreover, Shinohara et al. (2019) showed the effectiveness of applying the spatial learning method to full-waveform lidar data using a full-waveform network auto encoder (FWNetAE), consisting of a PointNet [7] based encoder and a naïve multi-layer-perceptron based decoder [27]. FWNetAE [27] employs PointNet [7] in its encoder, which can input even spatially irregular data and directly handle waveforms and coordinates (the first peak return) associated with the waveforms, despite the fact that deep learning methods are usually used for regularly arranged data, such as images, audio, and text. The PointNet [7] based encoder enables spatial feature extraction, which was not possible with 1D CNN. This learning method using an auto encoder is called representation learning [28], which allows models to extract features from the input data. This trained encoder can extract a compact representation in the latent space of each spatial input data. In a comparison experiment between an encoder using PointNet [7] and an encoder using 1D CNN, we show that the encoder using PointNet [7] is more finely clustered in features than the 1D CNN method. However, the authors only showed the power of feature extraction with spatial input data using unsupervised representation learning [29], not the concrete results of supervised classification for waveforms. Furthermore, deep learning based semantic segmentation methods for waveforms without hand crafted feature generation or conversion to voxel or image have not been studied extensively.

**Figure 1:**
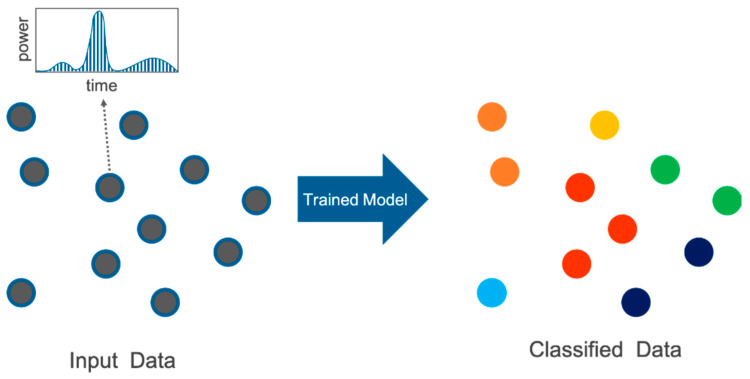
Overview of our proposed network (full-waveform network (FWNet)) for full-waveform light detection and ranging (lidar) data. Our FWNet predicts the class of each input data consists of waveform and its coordinate. The color of each point in the right figure is a class (land cover/land use).

In this paper, we show the effectiveness of spatial feature extraction in the semantic segmentation task for waveforms in a supervised manner. Our network is based on FWNetAE [27] using one of the typical architectures that can directly apply waveforms to representation learning tasks. We extended FWNetAE [27], where only unsupervised representation learning was possible, to directly predict the classes of waveforms in a supervised manner. Specifically, our model, namely the full-waveform network (FWNet), takes spatially distributed waveforms with associated geographical coordinates as input and predicts class vectors for each input in an end-to-end manner (Figure 1). Our primary contributions are as follows:

(1) Extending FWNetAE, which have only shown the effectiveness of unsupervised spatial representational learning on waveforms, we propose FWNet for supervised semantic segmentation and empirically showed that it outperformed previously proposed methods.
(2) Our FWNet can discriminate six ground objects (ground, vegetation, buildings, power lines, transmission towers, and street path) with high performance, merely using waveform and its coordinates without explicitly converting them into images or voxels in the semantic segmentation task.
(3) We experimentally demonstrated the effectiveness of the waveforms via an ablation study, which is an experiment to investigate whether or not each element contributes to the accuracy improvement when multiple elements related to the accuracy improvement are included in the proposed method, and the spatial learning method by visualizing the features extracted by the trained model.

This paper is organized as follows: Section 2 presents the literature on general deep learning methods and their application to 3D point clouds, as well as automatic analysis methods for full-waveform data. Section 3 discusses the approaches for the supervised learning of waveform and its coordinates data using a PointNet-based semantic segmentation network. Section 4 describes the dataset used for the experiment, shows the results for the test data, and provides a discussion on our proposed model via an ablation study and feature visualization. Finally, Section 5 presents a summary and conclusion.

## 2. Related Studies

### 2.1. Deep Learinig

In this paper, we focus on end-to-end supervised learning using deep learning without handcrafted features. A deep learning model has a set of functions called a layer and has a hierarchical structure of layers. The purpose of each layer is to extract features of the input data from the previous layer, by performing functions with nonlinear transformations and other functions, and send those data to the next layer. In particular, for image processing, trained deep learning models have been reported to match several features of the visual cortex [30,31]. This hierarchical feature extraction is also effective in the remote sensing domain using deep learning method [32]. The primary deep-learning-based method used in remote sensing is the convolutional neural network (CNN). CNNs are a type of deep learning method that can learn the features of the input data in a hierarchical and spatial manner. In each convolutional layer of the CNN, a learnable filter (kernel) extracts features by fusing spatial and channel information in the local receptive field. By using a series of convolutional layers with nonlinear activation functions and downsampling operators (max pooling or mean pooling), CNNs can generate robust feature that capture hierarchical patterns and global theoretical receptive fields. By performing convolutional operations on a region defined by the size of the kernel, spatial feature extraction is performed, and by performing further pooling, a wider range of information is aggregated into a single point. By repeating this operation, it is possible to extract features hierarchically from lower-level features, such as edges to higher-level abstracted features.

These deep learning methods have been widely used for 3D point clouds. The most conventional methods for 3D point clouds using deep learning are 2D CNNs that classify each pixel of 2D images projected from 3D point clouds [33,34]. These methods usually require the calculation of additional handcrafted features, that are an individual measurable property or characteristic of data, of point clouds (e.g., height, normal, height difference etc.) when they project 2D images from 3D point clouds. However, these methods are not used because of the information lost during the 3D projection onto a 2D image. More recent studies have used voxel data to represent 3D information [35]. Voxel-based methods use 3D convolutions for regular 3D grid data converted from the point cloud. In this case, when the point clouds are converted to voxels, the classification performance is adversely affected, because of information loss, because the GPU memory limit for learning the deep learning model makes it impossible to create high-resolution voxels, and the original information of the point cloud is lost [36]. To address these problems of the information lost in the translation process, a CNN-like network called PointNet was proposed to handle 3D point clouds. Additionally, some studies have applied CNN based techniques to irregular point clouds [37,38,39,40,41,42] after PointNet was proposed. These methods offer an integrated architecture that avoids high computational costs coming with high resolution voxels and allows point cloud data to be entered directly for semantic segmentation tasks.

Many researchers have investigated the deep learning-based methods for 3D point clouds acquired via ALS. For example, Yousefhussien et al. (2018) [37] proposed an FCN-based method. This method uses two input data, point clouds, and handcrafted features converted from 2D images. These input data are classified for each point using an end-to-end training process. Wang et al. (2018) [38] created a three-step pooling-layer method to classify point clouds. First, a point-wise features is extracted using a weight-shared MLP similar to PointNet [7]. Second, a spatial max-pooling layer is employed to extract features. Finally, another MLP layer is used to classify each feature. Wen et al. (2019) [18] proposed a multiscale FCN that considers direction. Winiwarter et al. (2019) [43] investigated the applicability of PointNet++ for not only benchmark data, but also actual airborne lidar point clouds. Additionally, a task-specific deep learning method for the extraction of ground information [44,45], and a tree species classification network were proposed [46].

### 2.2. Full-Waveform Data Analysis

Recently, full-waveform lidars have become the mainstream of airborne lidar measurement systems. The full-waveform lidar can record the reflection of the irradiated laser pulse from the object as a series of waveforms, representing the reflected intensity. Conventional airborne lidar record discrete peaks of intense reflection intensity, and there is a limit to the number of returns that can be recorded. Therefore, it can be said that the full-waveform data contains more information about the ground and surface than the conventional airborne lidar data. The researches on the generation of high-density point clouds (called Hyper Point Clouds), which cannot be obtained by the conventional airborne lidar, are carried out by using the measurement system of the full-waveform lidar [47].

Full-waveform data are highly advantageous for 3D point cloud classification tasks [48,49,50,51]. Moreover, full-waveform lidar data that include waveform provide rich information that easily discriminates some classes [52]. For example, a rule-based decision tree can be used for classification [53,54]. Other methods are based on machine learning with handcrafted features, such as support vector machine (SVM) classifiers [18], which offer a nonlinear classification method. SVM classifiers and other machine-learning methods have become widely utilized in point cloud classifications featuring some handcrafted features from full-waveform laser scanners [55,56,57,58,59]. Furthermore, for land use classification, Wang, C. et al. (2019) demonstrated the importance of not only the features from each waveform, but also the spatial features [60]. Additionally, Lai. et al. (2019) presented an ensemble method that uses the SVM model to improve classification ability [61], while some other papers use a multimodal method to combine hyperspectral images and waveform data [62,63].

Most of the above algorithms strongly depend on handcrafted features that are fed into statistical classifiers or simple machine learning algorithms. However, another data-driven approach was proposed by Maset et al. (2015) [25], who used the SOM to solve an unsupervised classification task on waveform within three classes (grass, trees, and roads) without handcrafted features. The same group presented an innovative method based on a convolutional neural network (CNN). The authors used these CNNs to solve a classification task with six classes (ground, vegetation, building, power line, transmission tower, and street path) for full-waveform lidar data [26]. Their proposed network includes a 1D CNN and a 2D Fully Convolutional Network (FCN). First, a simple 1D CNN is trained to predict the class of input waveform. The trained 1D CNN is used to preprocess each waveform to provide a class probability vector. In other words, the 1D CNN maps waveform into a compact representation. By leveraging the coordinates of the points associated with the waveform, the output vector generated by the trained 1D CNN, and height information are projected onto 2D grid data and subsequently labeled by the 2D FCN. The 2D FCN can easily take into account the spatial/positional and geometric relationships between adjacent data, as discussed for the semantic segmentation task for images. In Zorzi et al. (2019) [26], the local method for classifying the waveform separately was not effective, many models in the field of deep learning, such as image recognition and text translation, learn spatial information by performing global feature extraction as well as local feature extraction. The predictive ability of the 2D FCN suggests that the spatial learning method is advantageous for waveform. As a spatial learning method for waveform, an autoencoder-based representation learning method called FWNetAE was presented by Shinohara et al. (2019) [27]. Shinohara et al. (2019) can directly deal with spatially distributed full-waveform lidar data using a PointNet based encoder. FWNetAE [27] can input multiple waveform directly into the deep learning model by incorporating PointNet, which is capable of training point clouds, as opposed to the 1D CNN method [26], which trains waveforms independently. Spatial feature extraction can be achieved by FWNetAE for unsupervised learning. As a method of spatial learning, FWNetAE uses x, y and z coordinates and associated waveforms to extract the spatial features of each waveform within a certain range of neighborhood. The PointNet based encoder extracts the compact representation as a latent vector of each input data, and the decoder reconstructs the spatial distribution and waveform samples of the input data. Shinohara et al. (2019) demonstrated the effectiveness of the spatial learning method for waveform, but did not show the method’s specific classification abilities.

The remainder of our paper evaluates an end-to-end deep learning approach that uses PointNet based semantic segmentation architecture for spatially distributed waveform data, without any processes to convert those data to another data structure.

## 3. Proposed Method

The proposed method predicts the class probability of each waveform recorded from modern laser scanners. This paper describes the possibilities offered by deep learning for solving semantic segmentation tasks for waveform.

### 3.1. Problem Statement and Notation

The input datum for our network consist of geometric information and waveform. An input datum for the network is represented as a set of waveforms and coordinates associated with the waveforms (P) that forms an N×M matrix. N is the number of input waveform, and M is the dimension of waveforms and consisting of spatial dimensions (x, y, z), along with their waveforms. In addition, the waveforms are the data featuring intensity or power information in a time series.

**Figure 2:**
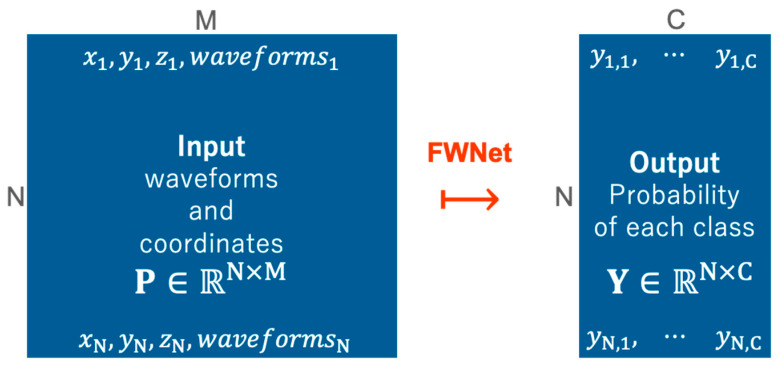
Problem statement in this study. Our model predicts the probability of each class ($Y\;\in \;{\mathbb{R}}^{N\times C}$) from the input data ($P\;\in \;{\mathbb{R}}^{N\times M}$) consisting of waveforms and coordinates (the first peak return) associated with the waveforms.

Figure 2 illustrates our goal, i.e., to teach the network to estimate the probability of each class as a vector over spatially distributed waveform as input data (${\mathbb{R}}^{N\times M}\mapsto {\mathbb{R}}^{N\times C}$). Here, C is the number of classes. Our FWNet is trained to predict the probability of each class $Y\;\in \;{\mathbb{R}}^{N\times C}$, corresponding to the input waveform, $P\;\in \;{\mathbb{R}}^{N\times M}$. The input to our method is not the entire analysis area (training or test data) at one time, but a small patch is clipped as the smallest unit and input to FWNet.

### 3.2. Proposed FWNet Architecture

**Figure 3:**
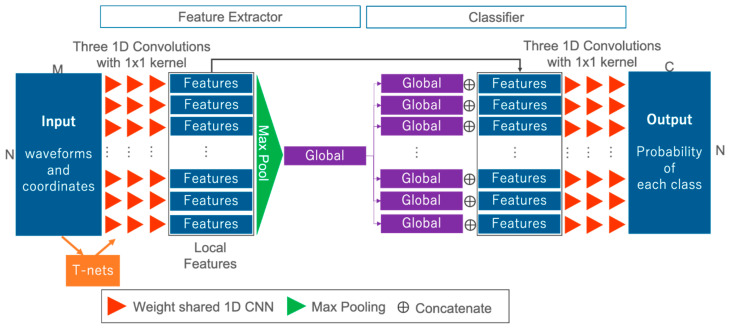
FWNet architecture. Input: waveforms and coordinates; output: class probability.

In this section, we present the network used to solve semantic segmentation tasks for waveforms. Figure 3 offers an overview of the FWNet. FWNet is characterized by the PointNet architecture, and transforms waveforms into the class vector Y, corresponding to the input data. In this study, the data (P) input into the network are waveforms with two dimensions (N × M); this represents a spatial distribution and geometric information and waveform. The PointNet-based feature extractor (the left side of Figure 3) contains three blocks.

The first block takes the input data defined by their geometric coordinates and the waveform and computes the local features for each point (as shown by the red triangle in Figure 3). The local features are added through 1D convolutional layers with a kernel size of 1 × 1:

(1) f(x)≈Activation(Wx+b)

where x∈X is an input datum for each layer, Activation is a nonlinear activation function with batch normalization, W is the learnable weight parameters, and b is biases. In this paper, we used three 1D convolutional layers with 256, 512, and 1024 filters, ending with the bottleneck layer of dimension 1024. Each layer is followed by a ReLU [64] as a non linier activation function and batch-normalization [65].

The second block comprises network transforms or T-nets used in PointNet [7] (as shown by the orange rectangle in Figure 3). T-nets make the points spatially invariable. T-nets estimate a 3 × 3 transformation matrix, which is applied to the input as a first step. T-nets add a transformation into a canonical space to roughly align point clouds to ease the following computation [7]. The t-nets consist of a multilayer perceptron (MLP), a max pooling operator, and two fully connected layers.

The third block computes the global features over all bottleneck layers (as shown by the green rectangle in Figure 3). To compute the global features, we use a max pooling layer as a symmetric function (i.e., the other word permutation-invariant function):

(2) $$f\left(\left\{x_{1},x_{2},\cdots ,x_{N-1},x_{N}\;\right\}\right)\approx g\left(\left\{h(x_{1}),h(x_{2}),\cdots ,h(x_{N-1}),h(x_{N})\;\right\}\right)$$

where x is the individual waveform with coordinate information, f denotes the function to be approximated, h is an individual input data-wise nonlinear transformation, and g is a symmetric function. A symmetric function is the function that produces the same output without any dependence on the input order, although there are input variations with N! when the number of input points is N. In this case, h is computed using a simple 1D convolutional operation, and g is the max pooling operation. Max pooling provides invariant features to the input order. To add global information into each of the local features, after the output of the max pooling layer, we concatenate each local feature and global feature (as shown by ⊕ in Figure 3). By using these three blocks, not only is feature extraction by independent one-dimensional convolution for each input point mad possible, but three-dimensional feature extraction is also possible.

We use the classifier for each feature to solve the semantic segmentation task (as shown on the right side of Figure 3). We use simple 1D convolutional operations as classification layers. After being transformed by three 1D convolutional operations, the class probability is estimated by the softmax layer:

(3) $$p_{i}=\frac{e^{y^{i}}}{\sum_{k=1}^{C}e^{y^{k}}}$$

where $p_{i}$ is the class probability of class i, $e^{y^{i}}$ is the final output value from the classification layer of each class, C is the total number of classes, and $e^{y^{k}}$ is the probability of class k. The final output of our FWNet is the probability of each class $Y\;\in \;{\mathbb{R}}^{N\times C}$, where N is the number of classes, and C is the number of classes.

FWNet aims to obtain the output $Y\in {\mathbb{R}}^{N\times C}$ from the input data, $P\in {\mathbb{R}}^{N\times M}$. Y is the probability of each class $p=\left\{p_{1},\;p_{2},\cdots ,\;p_{C-1},p_{C}\right\}$ corresponding to the input data. In the optimization process, we need to minimize the difference (error) between the ground truth $G\in {\mathbb{R}}^{N\times C}$ and the network output by minimizing the loss functions. In this study, we use cross entropy ($L_{CE}$) [66] as the loss function, which can be defined as

(4) $$L_{CE}=-\overset{N}{\underset{i=1}{\sum }}\overset{C}{\underset{j=1}{\sum }}G_{i,j}\log\left(Y_{i,j}\right)\;$$

where C is the total number of classes, $G_{i,j}$ means ground truth in class *j* as a one-hot representation, and $Y_{i,j}\;$is the predicted probability of class *j* in the softmax layer (Equation (3)).

Unlike the benchmarking data, the number of points in each class in the real-world point cloud data is highly imbalanced, which has an adverse effect on the final performance. We add the weight for the minor class to calculate $L_{CE}$. This weighted cross entropy ($L_{WCE}$) is defined as

(5) $$L_{WCE}=-\overset{N}{\underset{i=1}{\sum }}\overset{C}{\underset{j=1}{\sum }}W_{j}G_{i,j}\log\left(Y_{i,j}\right)\;$$

where $W_{j}$ is the weight for class *j*. The formula is defined as

(6) $$W_{j}=\frac{1}{\ln\left(1.2+\frac{a}{b}\right)}$$

where a is the number of points of the same category, and b is the number of all point clouds. In the training process, minimizing the loss function ($L_{WCE}$) is necessary. This minimizing loss function can be formulated as the following optimization problem:

(7) $$\underset{\theta }{\mathrm{argmin}}L_{WCE}\left(\theta \right)$$

where θ represents all the learnable parameters in our FWNet.

### 3.3. Model for Comparative Experiments

**Figure 4:**
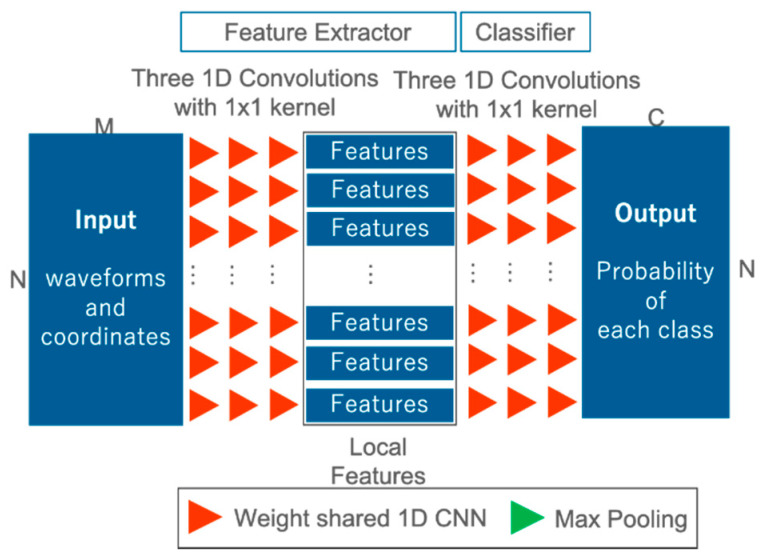
One-dimensional convolutional neural network (1D CNN) architecture (1D CNN Reproduce). Input: waveforms and coordinates; output: class probability.

In order to confirm the effectiveness of our proposed FWNet for spatial learning, we define an architecture design for comparison and conduct a comparison experiment. For the comparative experiment, we use a network corresponding to the 1D CNN proposed in Zorzi et al., 2019 [26], called 1D CNN Reproduce in this paper (Figure 4). 1D CNN Reproduce is only the local feature extraction in FWNet without T-nets and max pooling for the global feature. The hyper-parameters, such as the number of convolutional layers and the number of feature maps are set to the same values as FWNet. Furthermore, the optimization method and the loss function are the same as FWNet.

### 3.4. Model for Ablation Study

The use of waveforms, the application of segmentation models, and the implementation of class weights in the proposed FWNet are experimentally examined to see if each of them contributes to the estimation results. This effectiveness assessment is called the ablation study. Three models are defined for the ablation study.

**Figure 5:**
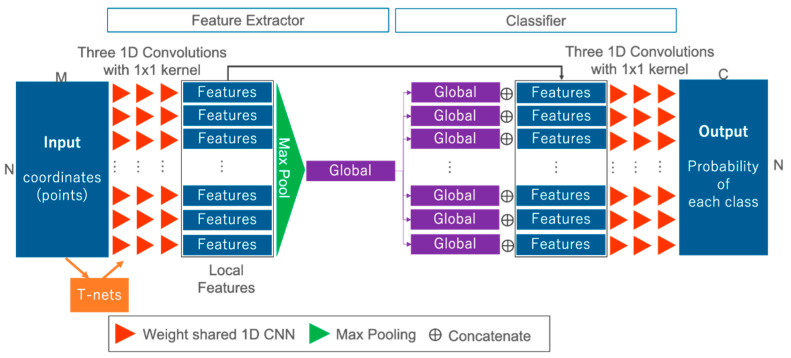
Geometry Model architecture. Input: Coordinates (point cloud) data; output: Class probability.

First, to determine the effectiveness of the waveforms, we trained the model with only geometry data (latitude, longitude, and height) without waveforms called the Geometry Model (Figure 5). This model is equal to naïve PointNet [7]. In other words, Geometry Model is mapped into the classification vector from the input point cloud data, ${\mathbb{R}}^{N\times 3}\mapsto {\mathbb{R}}^{N\times C}$, where N is the number of points.

**Figure 6:**
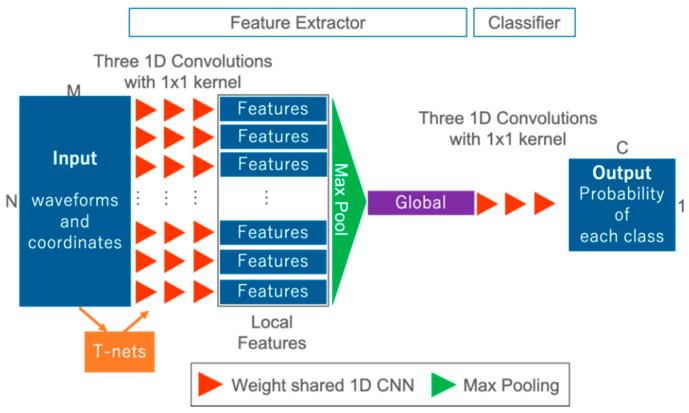
One Output Model architecture. Input: waveforms and coordinates; output: class probability.

Second, in order to confirm the ability of our FWNet, which can deal with many outputs that correspond one-to-one to many inputs, the One Output Model. This One Output Model is trained in the same dataset, but the final classification layer is changed to predict the probability vector of the only central point (Figure 6). In other words, the One Output Model is mapped into the classification vector from the input waveforms data, ${\mathbb{R}}^{N\times M}\mapsto {\mathbb{R}}^{1\times C}$, where N is the number of points, M is the dimension of each waveforms, and C is the number of classes. The following is a description of the One Output Model. The network design of the One Output Model has the same feature extractor as the FWNet shown in Figure 3, with the right half of the FWNet in Figure 3 removed. In other words, the class of the center is estimated from the waveforms data input from the global feature shown in Figure 6. In this One Output Model, the feature extractor part, which has the same structure as FWNet, uses the same settings as in Section 3.2. As shown in Figure 6, the One Output Model calculates the class probability by applying 1D CNN to the global feature. The loss function and hyperparameters are the same as those of FWNet.

Finally, to quantitatively evaluate the loss function that overcome imbalances the training data, we trained No Weight Model, which is the FWNet (Figure 3) using pure cross-entropy without weight (Equation (4)). The only difference from FWNet described in Section 3.2 is the loss function at the time of optimization, and the model architecture and hyperparameters are all the same.

### 3.5. Training Detail

The Adam optimizer [67] is used with an initial learning rate of 0.001, a momentum of 0.9, values of 0.5 and 0.999, and a batch size of 1. The learning rate is iteratively reduced based on the current number of epochs. The weights are initialized as described by Glorot et al. [68]. Our network was trained in PyTorch [69]. We used one “q node” of TSUBAME 3.0 [70], including one Tesla P100 graphics card.

### 3.6. Evaluation Metrics

The metrics for evaluating the test-data are recall, precision, and F1 score. These metrics are widely used to evaluate the performance of semantic segmentation tasks. Recall is an indicator of how many truly relevant results are returned, precision is an indicator of overdetection. The F1 score takes into account the precision and recall value and is generally appropriate when the categories are unevenly distributed.

The recall, precision, and F1 scores for each class are defined as follows:

(8) $$\mathrm{recall}=\frac{\mathrm{true}\;\mathrm{positive}}{\mathrm{true}\;\mathrm{positive}+\mathrm{false}\;\mathrm{negative}}$$

(9) $$\mathrm{precision}=\frac{\mathrm{true}\;\mathrm{positive}}{\mathrm{true}\;\mathrm{positive}+\mathrm{false}\;\mathrm{positive}}$$

(10) $$\mathrm{and}\;F1\;\mathrm{score}=2*\frac{\mathrm{precision}*\mathrm{recall}}{\mathrm{precision}+\;\mathrm{recall}}$$

where true positive is the positive data that were correctly classified, false negative is the positive data that were misclassified as negative, and false positive is the negative data that were incorrectly classified as positive.

### 3.7. Predictions

During the test stage, we used the patchwise prediction algorithm (Algorithm 1). The trained model directly handles all points in each small patch for semantic segmentation. We can merge the predicted class label from each small patch into the final prediction results. First, we loaded the test file. Next, we obtained the central points (query points) for nearest neighbor search (NNS) to make small patches with overwrapping. Next, we used the iterative prediction process shown below. (1) We made an input patch to train our model using NNS. (2) Our trained FWNet predicts the probability of a class for the input data. (3) We obtained the max value of the probability of a class. (4) We lastly put the predictions back into the original test file. Finally, we can get the test data with predicted class represented as predicted_classes in Algorithm 1.
**Algorithm 1** Prediction method for test data**Input:***test_data***Output:***predicted_classes***predict_Testdata**(*test_data*) **define**
*predicted_classes* = *test_data* central_points = **get_Centralpoint**(*test_data*) **for**
*i*
**in**
*len*(central_points)*input_data, index* = **get_ NNS** (test_data, lentest_data[i])*probability_vector* = **predict_Class**(input_data)*class_vector* = **get_Max** (probability_vector)*predicted_classes[index]* = *class_vector* **return**
*predicted_classes*

## 4. Experimental Results and Discussion

### 4.1. Dataset

**Figure 7:**
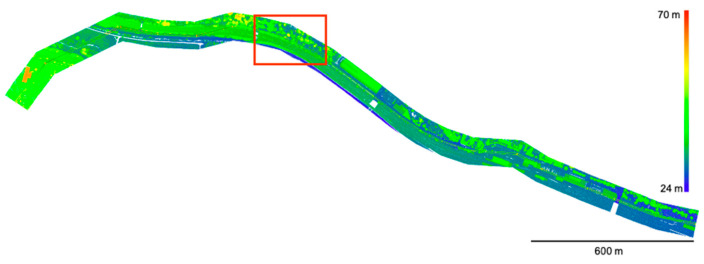
The training and test data used in this study. The red rectangle indicates the test area where we never used in the and parameter tuning process. The colors represent height values of the points from 24 m to 70 m.

**Figure 8:**
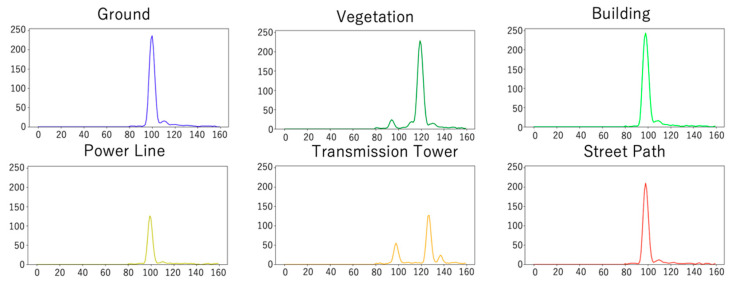
Example of waveforms used in this study. Each visualized waveform is the average value of 100 values randomly extracted from the classes.

Figure 7 shows the training and test data used in this paper, which were provided by Zorzi et al. (2019) [26]. The dataset was observed using an ALS (Riegl LMS-Q780 [71]) with a full-waveform laser scanner. This dataset consists of three pieces of information associated with each measured point: geometry, waveforms, and class labels. Geometry refers to the three-dimensional coordinates of a point represented by latitude, longitude, and height. The waveforms are described by 160 values. If the waveforms are shorter than 160, the value is padded with zeros to this length. The label indicates the class to which the point belongs. These labels were assigned from the six classes identified manually. The labels were manually assigned from six classes: ground, vegetation, buildings, power lines, transmission towers, and street path (Table 1). As the label indicates, the study area includes both natural surfaces and man-made objects. As shown in Figure 8, ground, building, and street path have similar waveforms with one strong peak. The vegetation and transmission tower have many peaks, while the power line has one weak peak.

In this study, training data were recorded for 8 million points. We used the training data points to create small patches to optimize the parameters. The following discusses how these patches were created. First, the *x, y* coordinates were randomly selected for the query point. Next, the nearest neighbor search (NNS) was used for the selected query point to obtain the surrounding *k* points and was incorporated as the input data for FWNet. Then, the value of *k* was determined such that the input data would have 8192 points. This value of *k* is dependent on the hardware environment in the training and prediction processes. A large *k* allows one to gather contextual information from a wider area, but it requires a high amount of GPU video memory compared to a small *k*. We determined the value of k to be the maximum value that can be used in the experimental environment.

The dataset used in this experiment was constructed by performing these procedures multiple times. The training and validation data were separated to avoid duplication, and we then used 5-fold cross validation. The cross validation was further divided into “training for cross validation“ and “validation for cross validation” in a ratio of 8:2 for the training data in Zorzi et al. (2019) [26]. Additionally, this dataset includes the test area (as shown by the red rectangle in Figure 4) used by Zorzi et al. (2019) [26]. We never used this test dataset in the training and validation process (including parameter tuning and determining the network architecture).

### 4.2. Classification Result

To quantitatively evaluate the classification results using our trained model, we calculated the recall, precision, and F1 score of each category and listed the results in Table 2; the average of the six classes calculated from each metric is shown in the column mean in Table 2. The classification result using our trained FWNet is shown in the row *FWNet* in Table 2. Each metric shows the mean value of the classification results from 5-fold trained models. The proposed model obtained F1 scores greater than 0.6 for five of the categories except for the transmission tower class. The class of transmission tower was a minor class with only 0.2% of the total training data, so we determined that the weighting for the loss function alone could not be enough. However, our model had a spatial feature extraction function to obtain contextual information, which enabled our model to correctly classify the ground, building, and street path with similar geometric features. Compared to 1D CNN using the individual learning method for waveform (1D CNN [26] in Table 2), our method offers higher performance for its recall values, except for the power line class. For classes with a large area, such as ground and building and street path, our proposed FWNet can classify simply by adding a wide range of global information to the local feature. On the other hand, we considered that global information makes a classification difficult for classes that exist in a narrow range, such as power line and transmission tower, because the local feature information contributes to the classification result. Moreover, we examined the reproduced 1D CNN model, as shown in Section 3.3. This reproduced model was trained on the same dataset used in FWNet. Our FWNet offers a classification ability higher than that of the reproduced experiment (1D CNN Reproduce in Table 2). We demonstrated applying the spatial learning method to waveforms in a semantic segmentation task using our FWNet.

**Figure 9:**
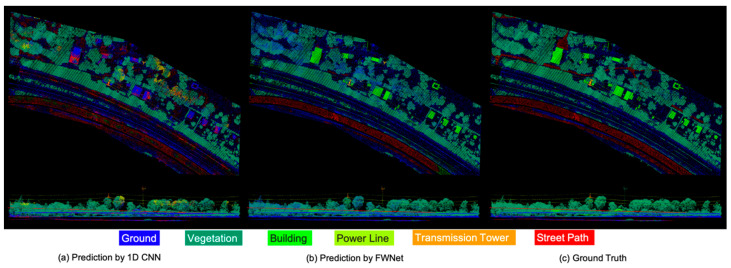
Predicted results of the test data.

The final predicted results of trained our FWNet are shown in Figure 9b. Compared to the ground truth shown in Figure 9c, the proposed FWNet model successfully predicted the correct labels for most of the visualized points in the test data. In Figure 9b, the final semantic segmentation results of the test data are shown. Qualitatively, our network tended to fail when classifying a street path (represented as red points) under the vegetation area (represented as green points) into the ground (represented as blue points). A typical misclassified area is shown as a circle in Figure 4. This misclassification likely occurred because of the similar geometric and radiometric waveform characteristics of the street path and ground in Figure 8. Furthermore, the 1D CNN Reproduce model shown in Figure 6a is severely misclassified as the building classes shown in green are classified as roads shown in red. A comparison with the results of the 1D CNN have shown that our proposed FWNet is effective in classifying ground objects with similar waveforms.

Our method achieves high performance classification of waveforms, which has not been effectively utilized in the field of deep learning and computer vision. With the establishment of a high-performance analysis method for ALS data, it is expected to be applied to the automatic generation of wide-area land use maps, as well as to the generation and updating of 3D maps for autonomous driving.

### 4.3. Ablation Study

We evaluated our proposed model with different settings, which were shown in Section 3.4, on the same dataset shown in Section 4.1. Table 3 represents the performance of the different models. First, we compared FWNet with Geometry Model to see if the use of waveform affected the estimation results. The row Geometry Model in Table 3 shows the results of the classification of the test data. Our model predicted classes with greater performance than Geometry Model for the test data. The precision values of each class for the Geometry Model test data were 0.52 for ground, 0.96 for vegetation, 0.96 for building, 0.85 for power line, 0.20 for transmission tower, and 0.77 for street path. Compared to the FWNet precision shown in Table 2, we observed the tendency of overdetection using the geometric information alone. The comparison results suggest that even in the conditions that are difficult to judge by geometry alone, the use of waveforms makes classification easier. In this way, we can show the effectiveness of the waveforms.

Next, we compared our FWNet with semantic segmentation model and One Output Model, as shown in Section 3.4. The prediction result of One Output Model is shown in the row Model B in Table 3. This result produced the highest mean precision value, but it took about 13 h to predict all of the test data using One Output Model. The reason for this time-investment problem was the use of point by point predictions with NNS sampling for every test point. Searching the kd-tree for the nearest neighbor of all N points has O(NlogN) complexity with respect to the sample size; using only a small amount of NNS sampling was effective for faster predictions. Our semantic segmentation model with the patchwise algorithm predicted classes faster than One Output Model.

Finally, to quantitatively evaluate the loss function that imbalances the training data, we trained the No Weight Model, which is the same model as FWNet using pure cross-entropy without weight (Equation (4)). This imbalanced classification result is shown in the row No Weight Model in Table 3. Compared to the weighted model shown in the columns FWNet, weighted cross entropy offered high performance for all metrics. Notably, the No Weight Model provided low recall values in the minor class. For example, the power line was 0.00, the transmission tower was 0.00, and the street path was 0.13. In this way, we demonstrated that the weighted loss function with class ratios is effective for use with highly imbalanced data sets.

### 4.4. Effects of Spatial Feature Extraction

To evaluate the quality of the spatial features extracted by our FWNet, we compared the 1D CNN (shown in Section 3.3), FWNetAE [27], and our FWNet. To compare the power of the feature extraction, we visualized the feature vectors of the test data we extracted from the trained 1D CNN, FWNetAE, and FWNet. For visualization purposes, t-distributed stochastic neighbor embedding (t-SNE) [72] was used to obtain the latent space in$\;{\mathbb{R}}^{2}$. The t-SNE parameter “perplexity” was set to 50. We visualized 100 randomly selected data in each class from the test data.

**Figure 10:**
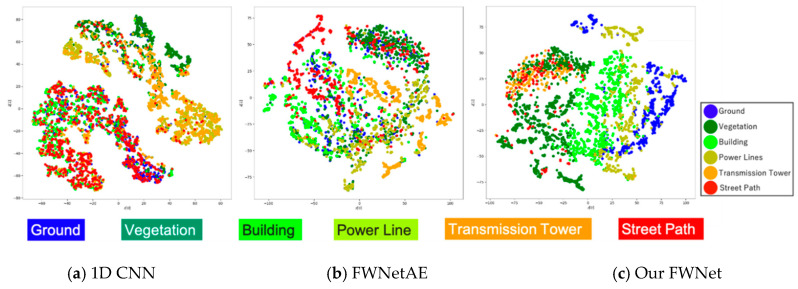
Latent space visualization and t-distributed stochastic neighbor embedding (t-SNE) projection of the feature vectors from the input data projected on a 2-dimensional space. We used the same 100 randomly selected data in each class from the test data for the three models.

First, we showed that the visualization result of our FWNet and 1D CNN. The feature vectors used in the visualization were the layers used to perform the classifications. For the FWNet model, the feature vector was observed to cluster consistent with the prior distribution (Figure 10c). The feature vectors generated from our FWNet provided a latent space where the clusters were more clearly separable than the 1D CNN model (Figure 10a). However, our model did not map waveforms into latent space with class-wise small clusters. To address this latent space separation, we considered a regularizer or penalty function that our model extracts the similar features of each class. Moreover, a large reception field is needed to more clear latent space, because we could only know the street path or ground from relative spatial information.

Second, we compared the unsupervised FWNetAE model and our supervised FWNet model. The feature vectors used in the visualization were the bottleneck layer of the trained encoder. The features were extracted by FWNetAE [27] and trained with the same dataset, but the label information was omitted. FWNetAE shows the tendency to separate each class in a latent vector without supervised learning (Figure 10b). This means that the spatial feature extraction was very effective for waveforms. However, the latent space was observed to be mixed in some classes other than our supervised model. Thus, we considered the task-specific method to be effective.

Generally, A comparison with FWNetAE, which is a supervised and unsupervised learning model using 1D CNN, shows that our FWNet is able to separate features in latent space, but it is still confirmed that features obtained from the learned model are still mixed. This is due to the two limitations of our model. The first limitation is that the PointNet-based model could not extract features at certain resolutions, despite deep learning methods achieve high performance by hierarchically extracting features from a wide range of information. To overcome this limitation, we will consider some recent hierarchical deep learning models with large receptive fields. A data structure of waveform and its coordinate was used for the geometric data. Considerable effort has been devoted to training geometric data by applying deep-learning techniques [73]. Recent deep-learning methods for geometric data are divided into metric-space-based or graph-based networks. One of the metric-space-based methods is PointNet++ [74]. PointNet++ was improved from PointNet to extract features hierarchically and obtain large receptive fields via a downsampling process in metric space. Meanwhile, the graph-based method [75,76] uses a graph convolutional operation for point clouds. In addition, some recent studies have shown the importance of global contexts when applying these methods to semantic segmentation tasks [77,78,79]. The second limitation is that we cannot handle waveform specific features. Waveform is defined as sequential data consisting of observed times and the power of returns. To learn time series data, recurrent neural networks, long short-term memory, and attention mechanisms are widely used.

## 5. Conclusions

This paper presented an end-to-end semantic segmentation model for spatially distributed waveform and the coordinate information associated with the waveform data observed from an aerial laser scanner (ALS). The potential difficulties in analyzing not only waveforms but also general ALS data using deep learning are that the data are spatially irregularly scattered. We have addressed this potential difficulty with the PointNet-based deep learning approach. Our FWNet used a PointNet based architecture that deals with waveforms and its coordinate even if the input data is irregular. The results demonstrated high classification performance for the invisible test data compared to the 1D CNN-based methods for waveforms. Specifically, our FWNet achieved a mean recall of 0.73, a mean precision of 0.81, and a mean F1 score of 0.76. Additionally, the results of this three-ablation study show the effectiveness of our semantic segmentation model. Moreover, FWNet presented a more meaningful feature vector than the 1D CNN-based individual classification model and the unsupervised autoencoder-based FWNetAE.

In future studies, to overcome the limitations discussed in Section 4.4, a more complex network will be considered, such as a metric space-based model or a graph-structured model, to examine more effective features on waveform compared to spatially irregular data. Additionally, we will consider the waveform awareness operation to extract rich features of sequential value.

## Figures and Tables

**Table 1:** Number of points for the training data and test data.

Class	Train		Test	
	Number of Data	%	Number of Data	%
Ground	1,787,352	20.4	193,070	18.1
Vegetation	4,719,634	53.9	765,327	71.7
Building	1,514,486	17.3	49,138	4.6
Power Line	71,978	0.8	8151	0.8
Trans. Tower	32,008	0.4	1829	0.2
Street Path	633,606	7.2	49,580	4.6

**Table 2:** Prediction results of the test data. Three metrics, recall, precision, and F1 score, were used. The bold text indicates the best performance. The 1D CNN ref is the result of reference [26], 1D CNN Reproduce is our experimental result using 1D CNN, and FWNet is our proposed method.

Method	Metrics	Ground	Veg.	Build.	Power Line	Trans. Tower	Street Path	Mean
1D CNN [26]	Recall	0.07	0.79	0.13	0.91	0.42	0.56	0.48
	Precision	-	-	-	-	-	-	-
	F1 score	-	-	-	-	-	-	-
1D CNN Reproduce	Recall	0.68	0.83	0.02	0.80	0.36	0.58	0.55
	Precision	0.51	0.96	0.14	0.26	0.05	0.29	0.37
	F1 score	0.59	0.89	0.04	0.40	0.08	0.38	0.40
FWNet	Recall	0.91	0.85	0.83	0.84	0.48	0.62	0.73
	Precision	0.56	0.97	0.95	0.92	0.61	0.94	0.81
	F1 score	0.69	0.91	0.88	0.88	0.53	0.75	0.76

**Table 3:** Ablation study on different components of our proposed method. The metrics in this table, recall (mRecall), precision (mPrec.), and F1 score (mF1), are the mean values. The boldface text indicates the best performance. The Time indicates the time required to predict the test data.

Method	FW	All Points	Weight	mRecall	mPrec.	mF1	Time(h)
*Geometry Model*	-	✔	✔	0.68	0.71	0.65	1
*One Output Model*	✔	-	✔	0.63	**0.77**	0.62	13
*No Weight Model*	✔	✔	-	0.28	0.28	0.27	1
FWNet	✔	✔	✔	**0.73**	0.73	**0.73**	1
